# Sub-lethal Doses of Polybrominated Diphenyl Ethers, in Vitro, Promote Oxidative Stress and Modulate Molecular Markers Related to Cell Cycle, Antioxidant Balance and Cellular Energy Management

**DOI:** 10.3390/ijerph16040588

**Published:** 2019-02-18

**Authors:** Simona Manuguerra, Cristóbal Espinosa Ruiz, Andrea Santulli, Concetta Maria Messina

**Affiliations:** 1 Department of Earth and Sea Science, Laboratory of Marine Biochemistry and Ecotoxicology, University of Palermo, Via Barlotta 4, 91100 Trapani, Italy; simona.manuguerra@unipa.it (S.M.); cristobal.espinosaruiz@unipa.it (C.E.R.); andrea.santulli@unipa.it (A.S.); 2 Marine Biology Institute, Consorzio Universitario della Provincia di Trapani, Via Barlotta 4, 91100 Trapani, Italy

**Keywords:** PBDEs, biomarkers, metabolism, oxidative stress, proliferation

## Abstract

In the present study, we evaluated the effects of different concentrations of the polybrominated diphenyl ethers (PBDEs) BDE-209, BDE-47 and BDE-99, on the vitality and oxidative stress of a HS-68 human cell culture exposed to the compounds for three days. The results showed that for this exposure time, only the highest concentrations produced a significant vitality reduction and oxidative stress induction (*p* < 0.05), measured as reactive oxygen species (ROS). Subsequently, in order to verify the effects of sub-lethal doses, cells were exposed for a longer time and data collected, after 12 and 20 days, to study ROS production and some molecular markers related to cell cycle and stress (p53, pRB, PARP, c-Jun and c-Fos), antioxidant status and proliferation (ERK, c-Jun and c-Fos), energy balance (NRF2, AMPK, HIF). Most of the biomarkers were influenced by the treatments, indicating that sub-lethal doses of PBDEs, for longer time, can enhance the production of ROS, altering the energetic metabolism, cell cycle and antioxidant balance, determining possible negative effects on the cell proliferation equilibrium.

## 1. Introduction

Polybrominated diphenyl ethers (PBDEs) are a class of organobromine compounds widely used as flame-retardant additives in a variety of consumer products such electronic devices, furniture, foams, plastics, and textiles [1] to reduce the risks of ignition and burning [2]. The chemistry of these compounds leads makes them stable for many years in the environment [3], where they are ubiquitous and persistent [4,5,6,7,8,9,10,11,12]. In fact, these compounds can be found both in the terrestrial and marine environment, as complex mixtures of numerous congeners [13]. They have been reportedly found in sediments, marine mammals, fish, bird eggs and also in human milk, serum, and adipose tissue [14]. The most abundant compounds found in the environment are 2,2′,4,4′-tetrabromodiphenyl ether (BDE-47) and 2,2′,4,4′,5-pentabromodiphenyl ether (BDE-99) [15,16], being dietary food and indoor dust the major sources of human exposure [17]. Some flame retardants, such a penta- and octabrominated diphenyl ether, have been prohibited in the USA and European Union [18,19,20,21]. The effects of PBDEs have been evaluated both in vivo than in vitro [22,23,24,25], in many experimental model systems with different approaches. However, the common evidence is that these compounds can exert a pattern of negative effects at cellular and molecular levels, varying from toxicity, to impairment of immune, reproductive and neurological system, to endocrine disruption and, also, cancer [2,26,27]. In living organisms, PBDEs may be metabolized by phase I and phase II detoxification enzymes, that render these compounds more soluble in bile or urine, as verified in exposed mice and rats; the most common phase I reaction is hydroxylation, caused by cytochrome P450 enzymes, while the mostly representative phase II reaction is conjugation with glutathione [28,29]. At a molecular level, it has been reported that PBDEs induce thyroxine-like and estrogen-like activity in vitro [30], as well as cell proliferation in MCF-7 breast cancer cells [31,32,33], in OVCAR-3 and normal ovarian CHO cells, by the induction of the G2/M or S phase of the cell cycle [34], at low doses (10^−12^ to 10^−9^ M). Furthermore, in Neuro-2a cells, BDE-47 was shown to increase the expression of p53 and p21, which down-regulate the expression of cyclin D1 and CDK2, and inhibits retinoblastoma protein (pRB) phosphorylation [35], although the mechanism(s) that trigger the process remain unclear.

Within the wide range of effects induced by several classes of PBDEs, oxidative stress is recognized as a common event, both in vitro and in vivo [36,37]. This process, evidenced by an overproduction of reactive oxygen species (ROS) with respect to the intrinsic antioxidant defenses, can be deleterious for all the cellular components and can influence signal transductions that regulate both cell cycle/apoptosis, energy balance and metabolism, opening new pathways that can induce cell transformation and cancer [38,39,40,41,42,43].

In view of these well-known roles of ROS in modulating the mentioned signal transductions pathways, our attention was directed toward the assessment, in PBDE-exposed cells, of some biomarkers related to oxidative stress and patterns of cell survive/apoptosis, cell cycle progression, cell metabolism and cancer. Among these, we monitored: 1) the protein p53, known as ‘the genome guardian’, that plays a main role in maintaining genetic stability integrity [44]; 2) the retinoblastoma protein pRB, a tumor suppressor that contributes to the cell cycle progression [45]; 3) the poly(ADP-ribose) polymerase (PARP), that is able to detect the DNA damage caused by many factors and permits the DNA repair [46]; 4) the extracellular signal-regulated kinase (ERK), also known as mitogen-activated protein kinase (MAPK), [47,48]; 5) c-Jun and 6) c-Fos, which are involved in oxidative stress-promoted apoptosis [49]; 7) adenosine 5′-monophosphate-activated protein kinase (AMPK), a serine/threonine protein kinase that serves as the most important energy sensor in the regulation of cellular metabolism [50]; 8) the hypoxia inducible factor (HIF), that regulates the central metabolism in relation to the oxygen availability, promoting also angiogenesis [51]; 9) the nuclear factor erythroid-2-related factor-2 (NRF-2), a redox sensitive transcription factor, which plays an important role in defending cells against oxidant stress [52]. In view of all these properties, the evaluation of these keys proteins could indicate the interference of PBDEs on cell homeostasis, energy management, antioxidant balance and cell cycle progression, giving indication on perturbation of standard pathways and functioning as an early warning system, to detect deviations that can compromise the normal life cycle, as we have reported in a recent study carried on a fish cell line [53]. Since most of the in vitro studies evaluated the effects of different PBDEs in acute exposure, in this study we also evaluated, in addition to a preliminary exposure to a wide range of concentrations for a short time, the effects of an exposure to sub-lethal doses, for a longer time, on biochemical changes.

## 2. Materials and Methods 

### 2.1. Cell Culture Maintenance, Treatment and Cytotoxicity Assay 

A human fibroblast cell line HS68 (ECACC n° 89051701, Sigma^®^, (Sigma-Aldrich, Saint Louis, MO, USA) was cultured in 75 cm^2^ plastic flasks (Nunc, Darmstadt, Germany), in Dulbecco’s Modified Eagle’s Medium (DMEM), supplemented with 10% fetal bovine serum (FBS), 2 mM glutamine, 100 i.u. mL^−1^ penicillin, 1% of non-essential amino acids and 100 mg mL^−1^ streptomycin (all reagents from Sigma-Aldrich, Saint Louis, MO, USA). Cells were grown at 37 °C with 5% CO_2_ under an 85% humidity atmosphere. Cells at 80% of confluence were detached with trypsin solution (0.05% of trypsin in PBS, pH 7.2–7.4) and pelleted by centrifugation (1000 rpm, 10 min, 25 °C). The cell suspension in completed medium was dispensed in a 96 multiwell plate at a density of 8000 cells/well and incubated, as described above, for 24 h before the exposure to the compounds. The PBDEs standards were provided by SPECTRA (Rome, Italy); stock solutions were prepared by dissolving the powder compounds in dimethyl sulfoxide (DMSO) and then diluting in complete medium (final concentration of DMSO 0.1%), in order to realize the various concentrations: BDE-209 was tested from 0.25 to 2 µmol/L, BDE-47 and 99 from 1 to 100 µmol/L (BDE-209 was tested at lower concentrations respect to the others two compounds, due to its lower solubility [53]). Cells were then incubated for 72 h and the toxicity of the compounds were determined using the (3-(4,5-dimethylthiazol-2-yl)-2,5- diphenyltetrazolium bromide (MTT, Sigma-Aldrich) assay according to Mosmann [54], as reported in Messina et al. [54]. The results were expressed as viability percentage in respect to the negative controls (untreated cells). Cytotoxicity data were recorded at 24, 48 and 72 h. Treatment of cells with 0.1% DMSO alone is always done in our lab for each experiment, and the absence of the effects by the vehicle is well known [55,56], for this reason vehicle viability data are omitted in the result graps. After the individuation of the sub-lethal concentrations for each compound, the next experiments were done in order to assess, by immunobotting, some molecular markers related to the different biochemical patterns.

### 2.2. Evaluation of Intracellular ROS

For the evaluation of the ROS, HS68 cells were seeded in 96 multiwell plate for fluorescence and incubated with BDE 209, 47 and 99 at the same conditions described above for cytotoxicity. After 48 h of treatment, intracellular ROS were analyzed by the dichlorodihydrofluorescein-diacetate (DCF-DA, Sigma-Aldrich) method, as reported by Kang et al. [57] and under conditions specified by Messina et al. [55]. DCF-DA is oxidized to dichlorodihydrofluorescein (DCF) by ROS. Each well was exposed to 10 µL of DCF-DA in HBSS (5 µg/mL), incubated for 5 min at 37 °C to allow the oxidation of the DCF-DA and successively read on a spectrofluorometer (Cary Eclipse, Varian, Mulgrave, VIC, Australia), at a wavelength of 485 nm of excitation and 530 nm of emission. The results have been expressed as relative fluorescence/µg of total proteins (rf/µg tp).

### 2.3. Evaluation of Biomolecular Markers by Immunoblotting

For the evaluation of molecular markers related to the different biochemical pathways, a long term experiment, lasting 20 days, was carried out, for each compound, at only one sub-lethal concentration (1 µmol/L). This concentration was chosen as the highest concentration that, in acute exposure, did not result in significant changes in viability and production of ROS.

HS68 cells (6.400 cells/cm^2^) were incubated in a 25 cm^2^ flask (Nunc) with the three PBDEs at 1 µmol/L, plus a mix of the three PBDEs at the same concentration, and sampling for immunoblotting were done at 12 and 20 days. For each compound at two replicates were realized each sampling timepoint. After the treatment, the cells were recovered as previously described, incubated 30 min on ice in (1:4) lysis buffer (1% 4-nonyphenylpolyethylene glycol (NP-40), 0.5% sodium deoxycholate, 0.1% sodium dodecyl sulfate (SDS), Sigma-Aldrich) cocktail of protease inhibitors, and sonicated. Protein concentration was measured in total lysate, according to Lowry et al. [58]. Experiments were carried out in triplicate.

Equivalent amounts of proteins (20 μg) were loaded on pre-cast gel for SDS–polyacrylamide electrophoresis (SDS-PAGE, Bio-Rad, Hercules, CA, USA) and blotted using a Trans Blot Turbo Transfer System (Bio-Rad). The correct amount of protein loading was confirmed by Red Ponceau staining. Filters were used for protein detection by primary antibodies (AbI) specifics for p53, retinoblastoma protein (pRB, IF8), Extracellular signal-regulated kinase 1 (ERK1), c-Fos, c-Jun, phospho-AMP-activated protein kinase (AMPK), hypoxia-inducible factor (HIF), poly (ADP-ribose) Polymerases (PARP) and Nuclear factor (erythroid-derived 2)-like 2 (NRF2) (Sigma-Aldrich Saint Louis, MO, USA). The primary antibodies were diluted in buffer at the concentrations suggested by the company for each AbI. In relation to the origin of the AbI, the appropriate secondary antibodies were used (anti mouse or anti-rabbit, anti-goat secondary antibody), conjugated with horseradish peroxidase (GAR/M-HRP Bio-Rad). The signals originated by immunoreaction were detected using enhanced chemo-luminescent (ECL) reagents (Bio-Rad). Images were obtained, photographed and digitalized with Chemi-Doc XRS (Bio-Rad), and further analysed with Image Lab software (Bio-Rad). The showed results, for each protein, represent the mean value of three separate immunoblotting and were expressed as the fold increase of the target protein, for each treatment, vs. the control.

### 2.4. Image Acquisition

Cells incubated with different treatments were observed daily until 20 days using an inverted microscope Nikon Eclipse Ti-S (Nikon Instrument Inc., Melville, NY, USA) and images were obtained with a Nikon DS-L3 digital camera (Nikon Corporation, Tokyo, Japan) and the DS-L3 Digital Camera Controller acquisition software. Images represent HS-68 cells observed with phase contrast microscopy at 20× magnification.

### 2.5. Statistical Analysis

The results are expressed as Mean ± Standard Error of the Media (SEM). Homogeneity of the variance was analyzed by the Levene Test. Data were analyzed by one-way analysis of variance (ANOVA), and Tukey or Games-Howell post-hoc tests were performed in order to make a multiple comparison between experimental groups. Differences were considered statistically significant when *p* < 0.05. All the data were analyzed by the computer application SPSS for Windows^®^ (version 15.0, SPSS Inc., Chicago, IL, USA).

## 3. Results

### 3.1. Effects of PBDE on Cytotoxicity and ROS Production

**Figure 1:**
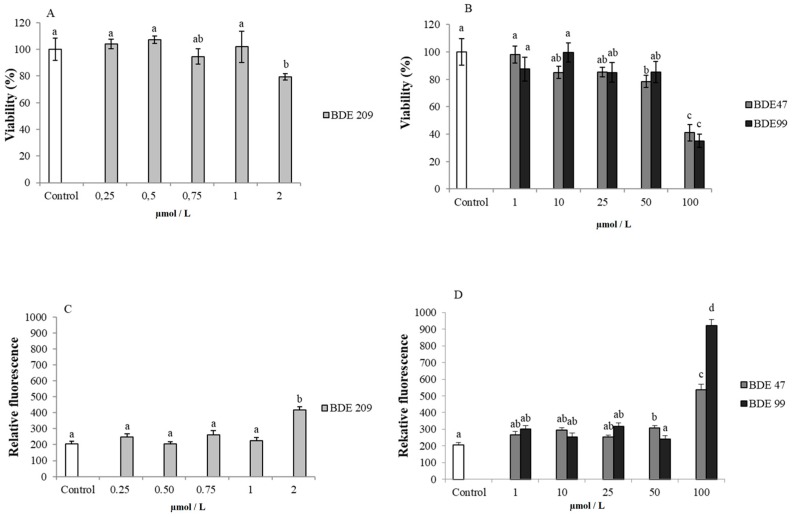
Cytotoxicity and oxidative stress on HS-68 cells exposed for 48 h to different concentrations of PBDEs: (**A**) vitality percentage (vs. control) of cells exposed to BDE 209 (0.25–2 µmol/L); (**B**) vitality percentage (vs. control) of cells exposed to BDE 47 and 99 (1–100 µmol/L).; (**C**) intracellular ROS production (expressed as relative fluorescence) on cells exposed to BDE 209 (0.25–2µmol/L) and (**D**) to BDE 47 and 99 (1–100 µmol/L). Bars represent the mean ± SEM (*n* = 6). Different superscript letters represent statistically significant differences (ANOVA; *p* ≤ 0.05) between groups.

The experiments were designed in order to firstly assess the response of cells, in terms of percentage of viability, to increased concentrations of PBDEs. In general, the results did not show a linear trend between increasing concentrations and mortality. Only the higher dose (for BDE 209 and BDE 99) and 50 and 100 µmol/L (for BDE 47) induced a significant cell toxicity, at 48 h (*p* < 0.05) (Figure 1A,B). The vitality results at 48 h were the most evident (data at 24 and 72 h not shown).

In cells exposed to the aforementioned concentrations of PBDEs, the presence of oxidative stress was verified by the measurement of ROS. After 48 h of incubation, all the treatments with the highest concentration of BDE 209 (2 µmol/L) and the highest concentration of BDE 47 and BDE 99 (50 and 100 µmol/L), presented an increased level of intracellular ROS, respect to the control samples (*p* < 0.05) (Figure 1C,D). 

**Figure 2:**
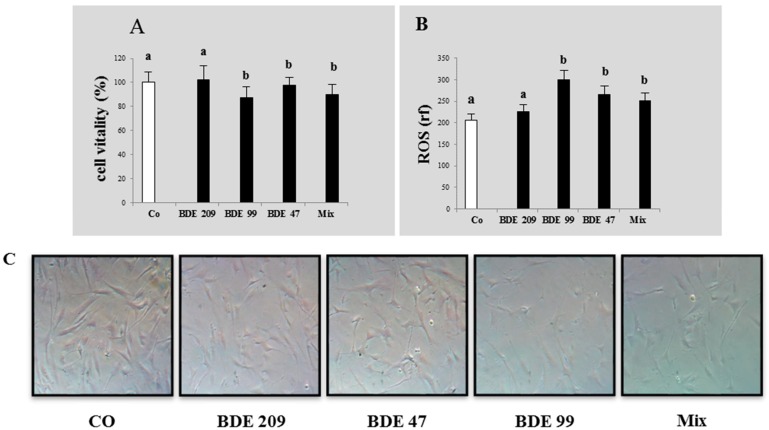
Cytotoxicity and oxidative stress on HS-68 cells exposed for 20 days to 1 µmol/L BDE 209, 99, 47 and MIX: (**A**) vitality percentage (vs. control); (**B**) intracellular ROS production (expressed as relative fluorescence). Bars represent the mean ± SEM (*n* = 6). Different superscript letters represent statistically significant differences (ANOVA; *p* ≤ 0.05) vs. control. (**C**) HS-68 cells after 20 days of treatment (phase contrast microscopy at 20× magnification).

After these experiments, we selected the sub-lethal dose of 1 µmol/L for the second part of the study, aimed to evaluate the effects of a long term exposure to low doses of PBDEs at 12 and 20 days, on some markers related to the different biochemical pathways. At the end of the experiment, we measured also the ROS production in cells treated with these sub-lethal doses and, differently from short term exposure slightly affected, the level of ROS resulted significantly increased in all treatments, respect to the control (*p* < 0.05) (Figure 2).

### 3.2. Effects of PBDE on Biomolecular Markers: p53, pRB, PARP

**Figure 3:**
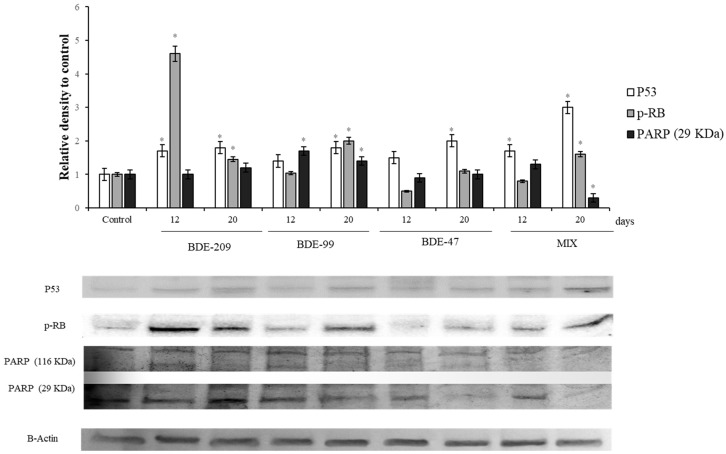
Immunoblotting of p53, pRB, PARP, evaluated on HS-68 cells exposed to 1 µmo/L BDE 209, 99, 47 and MIX for 12 and 20 days. Actin was used as internal control. The images are representative of at least three separate experiments. The relative protein quantification is represented in the graphic (* *p* < 0.05).

Figure 3 shows the levels of the protein p53 in cells exposed to 1 µmol/L of BDE-209, 99, 47 and MIX for 12 and 20 days. All the treatments presented an increase of the protein levels, respect to the control. The protein pRB (Figure 3) resulted significantly increased in cells after 12 days of treatment with BDE-209 and PBDEs mix (*p* < 0.05), while the treatment with BDE 99 and 47 caused a significant increase, respect to the control, only after 20 days (*p* < 0.05). After the incubation with BDE-209, 47, 99 and MIX, for 12 and 20 days, the levels of PARP changed significantly (Figure 3). In particular, cells treated with BDE-209 showed an increase of the proteolytic fragment at 12 days and 20 days (*p* < 0.05), while the cells treated with BDE 99 and mix of all, showed an increment of the fragment only after the 12 days.

### 3.3. Effects of PBDEs on ERK, c-Jun and c-Fos

**Figure 4:**
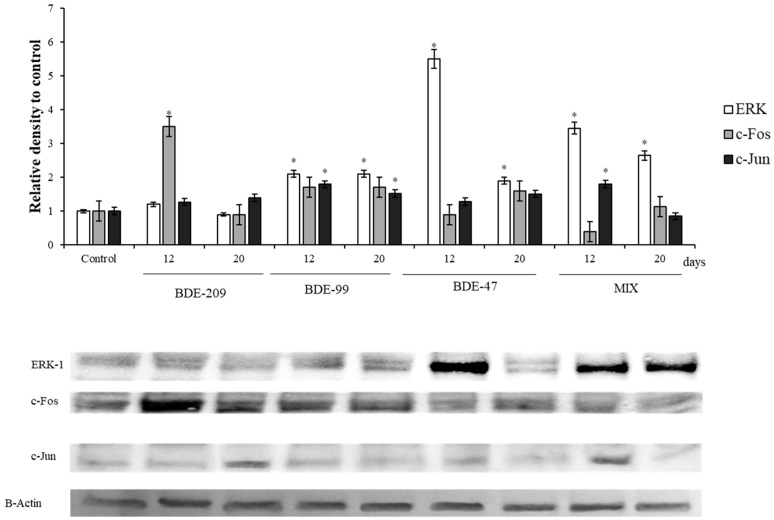
Immunoblotting of ERK, c-Jun and c-Fos, evaluated on HS-68 cells exposed to 1 µmol/L BDE 209, 99, 47 and MIX for 12 and 20 days. Actin was used as internal control. The images are representative of at least three separate experiments. The relative protein quantification is represented in the graphic (* *p* < 0.05).

The protein ERK1 and of some of its target, c-Jun and c-Fos, the main components of the AP1- complex, were also affected by the treatments (Figure 4). 

All compounds except BDE 209 and their mix displayed a significant increase of the protein levels, both at 12 and 20 days (*p* < 0.05).

### 3.4. Effects of PBDEs on NRF2, AMPK, HIF

**Figure 5:**
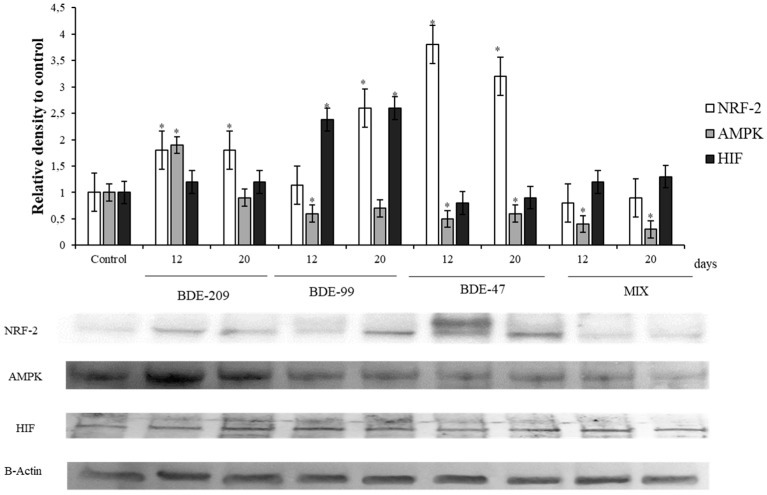
Immunoblotting of NRF2, AMPK, HIF, evaluated on HS-68 cells exposed to 1 μmol/L BDE 209, 99, 47 and MIX for 12 and 20 days. Actin was used as internal control. The images are representative of at least three separate experiments. The relative protein quantification is represented in the graphic (* *p* < 0.05).

The antioxidant master controller, NRF-2, showed a significant increase in HS-68 cells treated with BDE-209, 99 and 47, with respect to the control group, at 12 and 20 days (*p* < 0.05) (Figure 5); on the contrary, cells treated with the mix, exhibited the lower levels of this protein. Markers related to energetic balance were significantly affected by BDEs after 12 and 20 days of treatment: the sensor of the adenylate energy charge, AMPK, significantly increased in HS-68 cells treated with the BDE-209 after 12 days (*p* < 0.05), but not after 20 days. In addition, HS-68 treated with BDE-99, BDE 47 and MIX decreased significantly it levels after treatment, respect to the control (Figure 5).

The sensor of oxygen availability, the protein HIF, was affected by PBDEs treatments, in particular from BDE 99, that was able to produce a significant increase of HIF levels with respect to the control after 12 and 20 days (*p* < 0.05) (Figure 5).

## 4. Discussion

PBDEs have been commonly used as additive flame retardants in many industrial and commercial products such as furniture, textiles, polyurethane foam and plastics [1,2]. Unfortunately, PBDEs tend to bioaccumulate in water, soil, air, dust, sediments, animals, and tissues [59,60], exerting detrimental effects in all the investigated organisms, both in vitro and in vivo [52].

Regarding in vitro effects, it is reported that PBDEs can cause hepatotoxicity, immunotoxicity, reproduction impairment, apoptosis [61,62,63], endocrine-disrupting activity and carcinogenicity [64], as well as effects involving mitochondrial and endoplasmic reticulum stress, via oxidative damage [44,65].

As underlined by Wang et al. [64], although PBDEs have been banned in some countries and listed in the Stockholm Convention as Persistent Organic Pollutants (POPs) in 2009, the concentration of these compounds is still increasing in the environment at global level, through the food web, due to their lipophilic nature. Therefore, clarification of the mechanism underlying the toxic effects of PBDEs, especially at low doses, is mandatory.

In fact, as was reported in the study of Wang et al. [64], the exposures to realistic doses of PBDEs could determine a bimodal dose response in cellular systems, showing that low concentrations of BDE-47 could promote cell proliferation, while high concentrations of BDE-47 inhibited the cell growth. For these reason, in the present study, we performed a preliminary exposure to a range of concentration for each PBDEs to verify the effects on viability and ROS production. Then, we treated cells for a medium-long term with a sub-lethal dose of each compound, in order to investigate the effects on cell cycle, energy managements, stress and cell proliferation.

### 4.1. Effects of PBDEs on Cytotoxicity and ROS Production

Our results showed that exposure to increasing doses of these compounds did not determine a direct relationship between doses and viability reduction. In fact, only at the highest doses, cells showed a significant decrease of viability (Figure 1A,B) (*p* < 0.05). This phenomenon, yet observed in cells treated with PBDEs [64], was attributed to ‘hormesis’, that is the effect induced from ROS on stimulation of endogenous defense mechanisms, responsible of increase stress resistance and extend the life expectancy [66]. 

In general, our results are in accordance with Wang et al. [64], who showed a significant decrease of cell proliferation after treatment with BDE-47 using a range of concentration of 40–100 µmol/L for 3 days. 

A recent study on human monocytic leukemia THP-1 cell line showed that concentrations ranged from 3 to 25 µmol/L did not determine any significant decrease of vitality after 24 and 48 h [67]. Other authors found a significant decrease in cell viability after PBDE treatment, at lower concentrations, but this was dependent on cell systems, such as hepatoma and neuronal cells [35,66,68,69]. We found relevant differences in the literature related to PBDEs cytotoxicity, doses and cell lines, which pointed to BDE-47 as the compound that showed more effects at lower doses [35,37,68,70].

The hypothesis of a hormesis effect in this study is further supported by the increased levels of ROS observed in treated cells respect to the control (Figure 1C,D) both at 48 h in dose-mode and in long-term treatment, with sub-lethal doses (Figure 2). Although the effects of PBDEs on different cell lines have been showed to vary, our results agree with the previously described in hepatoma HepG2 cells and Neuro-2a cells, in which the treatment determined an increase of ROS [68] accompanied by a modulation of the Nrf-2 pathway [52]. 

It is well known that a prolonged situation of oxidative stress, an inadequate response to ROS production, as well as the exhaustion of antioxidant power, could affect cells’ homeostasis in different ways, stimulating cell transformations [71]. In fact, an intracellular increase of ROS has been described to induce DNA oxidation or damage, which allows the appearance of mutations, as well as the activation of proliferative processes involved in cancer initiation [71]. In this sense, cancer cells are characterized by the aberrant activation of oncogenes and deactivation of tumor suppressors, which promote their unrestrained proliferation. Nevertheless, the crucial prerequisite for proliferation is an energetic unbalance, as the genesis of cancer cell is associated with ‘metabolic transformation,’ a generally enhanced metabolism with several specific patterns serving for rapid and reckless acquisition of energy and metabolic building blocks [72,73,74,75,76]. 

### 4.2. Effects of PBDEs on p53, pRB, PARP

In the present study, regarding the cell cycle markers, the levels of protein p53 significantly increased after all PBDEs treatments, at 12 and 20 days (Figure 3). Under a stress situation, p53 regulates the cell cycle and inhibits retinoblastoma protein (pRb) phosphorylation, which produces the cell cycle arrest. This prevents the cells from entering in S phase and allows the repair of DNA, if needed. The cell cycle is a process that is strictly regulated by the ratio between positive and negative cell cycle regulatory factors, being p53 the heart of several fundamental cellular signaling pathways [76,77,78,79], and the most important of the induction of apoptosis and cell cycle arrest [80,81]. Interestingly, our data suggested that HS-68 cells increased p53 levels as it perceived a message of danger after PBDEs incubation, to induce the DNA repair and avoid the cell division. This result agree with others works, that reported an increasing of the p53 levels, after PBDEs exposure, in Neuro-2a cells, in spermatocytes from mouse [82] and HepG2 cells [68]. 

In our experiment, the protein pRb showed a significant increase after the treatment with the BDE-209, BDE-99 and PBDEs-Mix (at 20 days), although HS-68 cells, treated with BDE-47 for12 days, showed the pRb levels significantly decreased (Figure 3). Our data agree with those previously reported by Chen et al. [35] on Neuro-2 cells, that showed a decrease of pRb levels after BDE-47 treatment, but not for BDE-209. In the cell cycle, the G1/S-phase transition process is regulated by a series of checkpoints and restriction points [83], regulated by cyclin D-dependent kinases (CDKs)/cyclins complexes [84]. After active CDK is assembled in the nucleus, it phosphorylates the pRb [45]. Unphosphorylated pRb blocks the G1/S-phase transition by binding E2F transcription and by activate trans-repression [85,86]. Due to this, pRb dysregulations are involved in cancer cell transformation process, either due to absence of functional pRb [87], as well as overexpression, which has been described to be involved in tumour promotion of cells [88]. Regarding our results, either the increase or the decrease in pRb levels, reinforce the effects of PBDEs on cell cycle, although our data suggested these may be produced by different ways. 

As the p53 manages the decision between life and death, the PARP, located inside the nucleus, provides to detect and repair the DNA damage. Its activation (by proteolysis), is a cellular response to DNA damage caused by metabolism, chemicals or radiation [46,89]. Once PARP detects the damage, it binds to the DNA and let possible the mechanisms of repair, by adding units of poly-ADP-ribose [90]. In our study, the PBDEs treatment evidenced an increase of the PARP fragments, indicating its activation, a situation that require NAD as a substrate and that, gradually, induces a progressive decrease of ATP and the cell death [90]. On the other hand, PARP is inactivated by caspase-3 during apoptosis, which cleaves the PARP 116 kDa substrate into a stable 85 kDa fragment and a 25 kDa fragment [46,89]. The 25 KDa fragment, which is considered as an apoptotic marker [91], resulted significantly increased on HS-68 cells treated with BDE-209 and BDE-99 (Figure 3). These results agree with others studies in which BDE-209 [92], BDE-99 [93] and BDE-47 [94] increased the levels of cleaved PARP, inducing apoptosis on mouse fetal hippocampus neuron, HepG2 cell line and thyroid cells from Sprague-Dawley rats, respectively. 

### 4.3. Effects of PBDEs on ERK, c-Jun and c-Fos

In regard to PBDEs effects on cell proliferation, our results showed relevant variations on ERK, c-Fos and phc-Jun (Figure 4). One of the main pathways that integrates signals from the cell surface receptors to transcription factor is the Ras/Raf/MEK/ERK signaling cascade [47]. Extracellular signal- regulated kinase (ERK) is a vital component of the cascade and could be activated by the upstream molecule of this pathway [95]. The effects of the ERK-1 signaling pathway on the occurrence and development of various cancers have been reported [96,97,98,99]. In fact, when the phosphorylated ERK enters the cell nucleus, many transcription factors regulating cell cycle, cell proliferation and apoptosis are phosphorylated, like activator protein-1 (AP-1) [47,100]. The primary subunits of transcription factor AP-1 are conformed by ph-c-Jun and c-Fos, which are well-known proto- oncogenes, and plays an important role in cell proliferation and apoptosis [49]. In our experiment, ERK1, phc-Jun and c-Fos were increased after BDE-209, BDE-99, BDE-47 and Mix treatment (Figure 4), which agree with the previously reported results, that showed the induction of ERK protein by PBDEs treatment. The induction of ERK after BDE-47 treatment has been described in different models as human lung cancer cells [101] or invertebrate models as *Paracyclopina nana* [102]. Furthermore, prolonged ERK activity results in activation and stabilization of transcription factor AP-1 [103,104]. This, together with the fact of ERK1 via CDK4, as well as c-Fos, may produce pRb phosphorylation and degradation [104], may explain the increase of ERK1 and c-Fos and the decrease on pRb levels observed on HS-68 cells treated with BDE-47. Surprisingly, HS-68 cells treated with BDE-209, BDE-99 and mixture showed significantly increased pRb levels with respect to the control, which contrast with the increased ERK1 levels (Figure 3 and Figure 4). However, the high content of pRb protein, observed in cells treated with BDE-209, BDE-99 and Mix, could be partially explained by an energetic balance disorder, due to the relationship between the pRb phosphorylation and AMPK [105].

### 4.4. Effects of PBDEs on AMPK, HIF, NRF2

In this context, the stress situation caused by BDE-209, BDE-99 and Mix, could increase the requirements of ATP and oxygen, causing a certain impact on the metabolism. Indeed, our results showed a significant increase of AMPK in cells treated with BDE 209 as well as an increase of HIF levels on cells treated with BDE-209, BDE-99 and Mix (Figure 5). AMPK is an enzymatic complex activated by the increase of AMP/ATP ratio, so it is considered as a detector of the cell energy levels [106]. Previous studies have shown that AMPK is a central regulator of lipid metabolism and glucose homeostasis [106,107] stimulating energy-producing pathways [108]. HIF is a transcription factor involved on adaptive stress response that is sensitive to the decreases of oxygen availability in the cell environment, playing a pivotal role in the angiogenesis progress [109,110]. The increase of AMPK and HIF levels on cells treated with BDE-209, BDE-99 and Mix, suggests an evident effect on ATP and oxygen availability, which could justify the relevant increase of pRb on these cells, as we described above. Besides, this hypothesis is in concordance with the fact that HIF could activates pRb via RBP2 [109].

Concerning the effects of PBDEs on the oxidative status of HS-68 cells, our results showed a significant increase of NRF2 (Figure 5). NRF2 is a transcription factor of the cap ‘n’ collar basic region leucine zipper (cnc bZip) family, controlling the expression of various cytoprotective antioxidant enzymes. This transcription factor is found in many tissues, and is activated in response to a wide range of oxidative and electrophilic stimulation, including ROS and some chemical compounds [111]. Oxidative and electrophilic stress factors stimulate the release of NRF2 that upregulates the expression of NRF2/ARE-linked antioxidant and detoxifying genes. Our results agree with the previously reported on Neuro-2a cells treated with BDE-47, that showed a transcriptional induction of the *nrf2* gene and a significant increase in mRNA expression of the main antioxidant response genes in the NRF2 pathway [52]. Thus, NRF2 pathway in HS-68 cells also played a role in regulating the ‘protective effect’. In addition, the intracellular ROS production reinforce the hypothesis by which the PBDEs determine its toxic mechanism, mediating by oxidative damage, as we commented above. This observation could support also the results related to effect of PBDEs on the metabolism regulation, evidenced by the AMPK and HIF levels. 

Since the toxic mechanism of PBDEs has been suggested to be mediated by the oxidative damage [36,52,53,68,112], we hypothesized that low concentrations of PBDEs might also activate ‘adaptive responses’ through oxidative stress-related signaling such as NRF2 and metabolism adaptation by HIF modulation, that can promote proliferative pathways.

## 5. Conclusions

**Figure 6:**
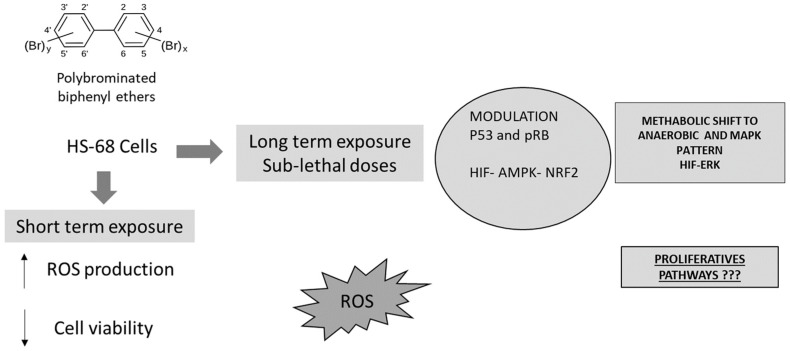
Proposed pathway explaining some biochemical effects, induced in vitro by PBDEs, on cell cycle, antioxidant status, metabolism and proliferation.

In conclusion, the present results show that PBDEs exposure affected the cells homeostasis in different ways. First of all, our results demonstrate that PBDEs, also at sub-lethal doses, induced oxidative stress, influencing the metabolism and the cell cycle (ATP, oxygen and NAD^+^ requirements). Concomitantly, BDE-209, BDE-99 and BDE-47 produced DNA damage, modifying some proteins involved on cell cycle and apoptosis modulation. The results also suggested that NRF2 played a central role in the cell defense against the PBDEs effects. These compounds induced its dangerous effects mainly by oxidative stress, affecting cell cycle, the energetic balance and finally representing a possible risk of cells proliferation and transformation (Figure 6).
